# Effects of Pumpkin Seed Cake in Rabbit Diets on Blood Indices, Oxidative Status, and Trace Element Distribution in Tissues

**DOI:** 10.3390/ani16091291

**Published:** 2026-04-22

**Authors:** Zuzanna Siudak, Dorota Kowalska, Anna Czech, Ewa Drąg-Kozak, Bożena Nowakowicz-Dębek, Kinga Szczepanik, Małgorzata Świątkiewicz, Sylwia Pałka, Paweł Bielański, Małgorzata Grzesiak

**Affiliations:** 1 Department of Small Livestock Breeding, National Research Institute of Animal Production, Krakowska 1, 32-083 Balice, Poland; dorota.kowalska@iz.edu.pl (D.K.); pawel.bielanski@iz.edu.pl (P.B.); 2 Department of Biochemistry and Toxicology, University of Life Sciences in Lublin, Akademicka 13, 20-950 Lublin, Poland; anna.czech@up.edu.pl; 3 Department of Nutrition, Animal Biotechnology and Fishery, University of Agriculture in Kraków, Al. Mickiewicza 24/28, 30-059 Kraków, Poland; ewa.drag-kozak@urk.edu.pl; 4 Department of Animal Hygiene and Environmental Hazards, University of Life Science in Lublin, Akademicka 13, 20-950 Lublin, Poland; bozena.nowakowicz@up.edu.pl; 5 Department of Animal Nutrition and Feed Science, National Research Institute of Animal Production, Krakowska 1, 32-083 Balice, Poland; kinga.szczepanik@iz.edu.pl (K.S.); malgorzata.swiatkiewicz@iz.edu.pl (M.Ś.); 6 Department of Genetics, Animal Breeding and Ethology, University of Agriculture in Krakow, Al. Mickiewicza 24/28, 30-059 Kraków, Poland; 7 Department of Endocrinology, Institute of Zoology and Biomedical Research, Faculty of Biology, Jagiellonian University, Gronostajowa 9, 30-387 Kraków, Poland; m.e.grzesiak@uj.edu.pl

**Keywords:** rabbit, pumpkin seed cake, blood parameters, oxidative stress, antioxidant status, trace elements

## Abstract

The focus of modern livestock production is increasingly shifting toward improving animal health, welfare, and product quality through the use of natural feed ingredients. Pumpkin (*Cucurbita* spp.) and its seeds are of interest because they contain biologically active compounds, including tocopherols and phenolic antioxidants. This study evaluated the effects of pumpkin seed cake (PSC) in rabbit diets on blood parameters, oxidative status, and trace element distribution in tissues. Sixty Popielno White rabbits were initially assigned to three dietary groups: control (0% PSC), 5% PSC, and 10% PSC. At 90 days of age, samples from 30 rabbits (10 per group) were collected and analysed. PSC supplementation significantly increased red blood cell count, haemoglobin, haematocrit, and platelet indices (*p* ≤ 0.05), indicating affected haematological status. It also reduced (*p* ≤ 0.05) urea, triglycerides, total cholesterol, and LDL cholesterol. Antioxidant status significantly improved, as indicated by higher superoxide dismutase activity and ferric-reducing antioxidant power, together with lower malondialdehyde levels (*p* ≤ 0.05). Mineral analysis showed lower manganese concentrations in muscle and kidney tissues; cadmium remained low, and lead was below the detection limit in muscle and liver samples. Overall, PSC may be considered a promising feed ingredient that supports haematological status, antioxidant protection, and metabolic balance under the conditions of the present study.

## Simple Summary

There is growing interest in using natural feed ingredients to improve animal health and reduce the use of conventional protein sources such as soybean meal. Pumpkin seed cake, a by-product remaining after oil extraction, contains protein and many natural compounds with antioxidant properties. In this study, we examined whether adding pumpkin seed cake to the diets of growing rabbits affects blood parameters, lipid profiles, antioxidant status, and mineral levels in tissues. The results showed that diets containing pumpkin seed cake affected haematological parameters, including higher red and white blood cell counts, haemoglobin, haematocrit, and platelet indices, together with changes in leukocyte profiles, lower blood lipids levels (total cholesterol, triglycerides, and LDL), improved selected biochemical parameters (including reduced urea levels), and enhanced antioxidant status compared with rabbits fed the control diet. The addition of pumpkin seed cake also reduced the concentration of manganese in muscle and kidney tissues. Lead was not detected in edible tissues; cadmium remained at low and safe levels. These findings indicate that pumpkin seed cake may be considered a promising feed ingredient that supports selected physiological and metabolic functions in rabbits under the conditions of the present study.

## 1. Introduction

Modern livestock production, including rabbits, increasingly emphasises animal health, welfare, and product quality [1]. In the search for natural alternatives to synthetic health-promoting agents, plant-derived feed materials with documented biological activity are gaining interest [2,3]. Among these, pumpkin (*Cucurbita* spp.) has attracted particular attention, as both its fruits and seeds contain a wide range of nutrients and bioactive compounds valuable for animal nutrition [4]. Pumpkin provides carotenoids, vitamins, polyunsaturated fatty acids, and phenolic compounds, which exhibit antioxidant, anti-inflammatory, and immunomodulatory properties [5]. These compounds exhibit multidirectional effects that support animal health, while improving the quality of animal products [6]. Different parts of the plant contribute distinct nutritional effects: pumpkin pulp supports gastrointestinal and metabolic functions [7], whereas the seeds constitute a richer source of protein, minerals and bioactive lipids, such as linoleic and oleic acids, known for their immunomodulatory potential [8]. Pumpkin seeds also contain phytosterols, tocopherols, cucurbitacins, and flavonoids, which contribute to their antioxidant and hepatoprotective activities [9,10,11,12]. The documented antiparasitic properties of pumpkin seeds are notable; they facilitate the elimination of internal gastrointestinal parasites, such as nematodes and tapeworms, which may reduce reliance on synthetic anthelminthic agents [11,12,13]. The immune-stimulating effect of some of the components contained in pumpkin seeds further promotes improved overall animal welfare [4]. Studies in poultry have shown that pumpkin seed oil can beneficially modulate lipid metabolism, reduce liver enzyme activity, and improve antioxidant status [14,15,16].

Reports on the effects of pumpkin on mineral metabolism in animals are limited, although existing studies suggest potential protective and detoxifying properties. For instance, in rats, pumpkin (*Cucurbita pepo*) fruit extract has been shown to mitigate lead-induced neurotoxicity, suggesting that pumpkin-derived components may have a regulatory influence on metal metabolism within the body [17]. As pumpkin seeds and their by-products have been shown to contain variable levels of essential trace elements and heavy metals, depending on environmental and agronomic conditions [18], it is necessary to evaluate their impact on mineral accumulation in animal tissues. Therefore, when introducing pumpkin seed cake (PSC) into rabbit diets, its physiological effects must be assessed alongside its safety profile, including its influence on tissue concentrations of manganese, zinc, iron, cadmium and lead, parameters crucial for both animal health and consumer safety. The assessment of heavy metals and trace elements was conducted to evaluate food safety and mineral homeostasis rather than meat quality traits.

Although PSC may partially replace conventional protein sources, such as soybean meal, in practical feeding systems, its effects on physiological and metabolic responses in rabbits remain insufficiently characterised. In particular, information on its influence on blood parameters, oxidative status, and mineral distribution in tissues is still limited. Therefore, the aim of this study was to evaluate the effects of dietary inclusion of pumpkin seed cake (PSC) on haematological and biochemical blood parameters, oxidative status, and the distribution of trace elements in selected rabbit tissues. Based on the documented bioactive properties of pumpkin seeds, we hypothesised that PSC supplementation may positively influence physiological status in rabbits.

## 2. Materials and Methods

### 2.1. Ethical Statement

All procedures involving live animals were approved by the First Local Ethics Committee for Experiments with Animals in Kraków, Poland (Resolution No. 721/2023). The experiment adhered to the institutional guidelines of the National Research Institute of Animal Production and complied with relevant Polish and EU animal welfare regulations. The health status of rabbits was monitored regularly by a veterinarian.

### 2.2. Animals

This was an in vivo feeding trial conducted on Popielno White rabbits to assess the effect of dietary pumpkin seed cake on haematological and biochemical blood parameters, oxidative status, and the accumulation of heavy metals and trace elements in selected tissues. A total of 60 Popielno White rabbits were randomly assigned at weaning (35 days of age) to three dietary treatments (*n* = 20/group): control (C)—0% pumpkin seed cake (*Cu-curbita pepo* L.) (PSC), 13% soybean meal; treatment 1 (T1)—5% PSC, 6.5% soybean meal; and treatment 2 (T2)—10% PSC, 0% soybean meal. Animals were allocated to experimental groups based on litter origin, sex, and similar initial body weight to ensure comparable starting conditions; assignment was random within these constraints. Rabbits were reared at the K-083 rabbit farm of the National Research Institute of Animal Production (Poland) from 35 to 90 days of age. After weaning, animals were housed in two-tier wire-mesh cages (60 × 80 cm), with four rabbits of the same sex per cage (five cages per treatment group). Males were housed on the upper tier and females on the lower tier. The stocking density complied with Polish legal welfare requirements [19]. The rabbits were maintained under natural gravitational ventilation, with an ambient temperature ranging from 14 to 18 °C and relative humidity from 50 to 65%. A photoperiod of 14 h light and 10 h dark (14L:10D) was applied. All zoo-hygienic and technological conditions were in accordance with national regulations for farm animal production [19]. Environmental enrichment was provided in the form of wooden gnawing blocks and elevated platforms. Animals had ad libitum access to feed and water. Feed intake was recorded per cage and recalculated per animal. The rabbits were included in a veterinary prophylaxis programme, including vaccination against myxomatosis and rabbit haemorrhagic disease (RHD). Animals remained clinically healthy throughout the experimental period. Initial body weights averaged 962 g (C), 973 g (T1) and 972 g (T2).

Organic PSC was obtained from a commercial crushing plant. Both PSC and the experimental feed mixtures were analysed using standard AOAC methods: dry matter (934.01), crude protein (2001.11), crude fat (920.39), crude fibre (978.10) and crude ash (942.05). The amino acid composition of the experimental diets was determined after acid hydrolysis (and oxidation hydrolysis for sulphur-containing amino acids) using ion-exchange chromatography with post-column derivatization, performed with an automatic amino acid analyser (AAA 500, INGOS, Prague, Czech Republic), according to AOAC procedures (994.12) and Commission Regulation (EC) No 152/2009. The digestible energy (DE) of the experimental diets was estimated based on their proximate composition using the equation proposed by Wiseman et al. [20]: DE (MJ/kg DM) = 12.912 − 0.0236 CF + 0.010 CP + 0.020 EE, where CP, EE, and CF represent crude protein, ether extract and crude fibre (g/kg DM), respectively. The detailed composition of the diets, chemical composition, and microelement concentrations of PSC, as well as the feed mixtures and amino acid composition of the diets, are provided in Table 1, Table 2, Table 3 and Table 4. The diets were formulated to be comparable in terms of crude protein content and to evaluate the effect of pumpkin seed cake inclusion. Although the diets were not strictly iso-energetic or iso-aminoacidic due to the replacement of soybean meal with pumpkin seed cake, they were balanced using standard feed formulation practices. Despite some differences in amino acid levels, key essential amino acids, such as lysine and methionine, were within, or close to, commonly recommended ranges for growing rabbits [21]. Furthermore, although the dietary crude fibre content was lower and the digestible energy content was higher than current recommendations, all experimental diets were consistent across treatments, allowing for a valid comparison of the dietary effects. The nutritional recommendations were based on standard feeding guidelines for fur animals [21].

At 90 days of age, 10 rabbits per group (5 males and 5 females) were randomly selected from the initial population of 60 animals, ensuring that body weights were within ±10% of the group mean. These animals were used for post-mortem analyses to obtain representative samples for haematological, biochemical, oxidative and mineral determinations. The individual animal was considered as the experimental unit for sampling and statistical analyses; each animal contributed one biological replicate. The remaining animals (*n* = 10 per group) were not included in post-mortem analyses but were used for growth performance and production-related evaluations throughout the experimental period, as reported in Siudak et al. [22]. After a 16-h fasting period with free access to water, rabbits were humanely stunned and slaughtered following Blasco et al. [23]. All animals were sampled under identical pre-slaughter conditions to minimise physiological variability. Peripheral blood was collected during exsanguination, while samples of longissimus lumborum muscle, liver, and kidney were collected post mortem, snap-frozen in liquid nitrogen, and stored at −80 °C. At slaughter, a total of 10 rabbits per dietary group (5 males and 5 females) were sampled for haematological, biochemical, and mineral analyses. Due to the limited availability of plasma of sufficient quality and volume after sample collection and processing, oxidative stress parameters were determined in samples obtained from 6 rabbits per group. All samples were collected strictly according to the predefined dietary treatments; each animal contributed one biological replicate to the respective analyses.

### 2.3. Blood Collection and Processing

Peripheral blood was collected during exsanguination at slaughter. Depending on the analytical purpose, blood was collected into EDTA tubes (haematology), tubes with clot activator (serum for biochemical analyses), or lithium-heparinized tubes (plasma for lipid profile and oxidative stress parameters). All samples were processed immediately after collection and centrifuged within 30 min; serum and plasma samples were stored at −80 °C until analysis. All blood samples were collected at the same time point during exsanguination to minimise variability related to circadian or post-mortem changes. None of the collected samples showed visible haemolysis; all were included in subsequent analyses.

### 2.4. Morphological Analysis of Blood

Whole blood was collected into EDTA tubes and analysed using a Mythic 18 VET haematology analyser (Orphee Medical, Geneva, Switzerland). Parameters included WBC, LYM, MON, GRA, RBC, HGB, HCT, MCV, MCH, MCHC, RDW-CV, RDW-SD, PLT, PCT, MPV, P-LCR and PDW. Procedures followed the methodology described by Szczepanik et al. [24] and the manufacturer’s instructions. Reference ranges were those provided by the reagent manufacturer.

### 2.5. Biochemical Analysis of Blood

Blood for biochemical evaluation was collected into tubes with clot activator, centrifuged (3500× *g*, 10 min, 4 °C), and serum stored at −80 °C. This centrifugation force is commonly applied in rabbit blood processing and yields clear serum suitable for biochemical analyses. Serum samples were analysed within a consistent storage period to avoid degradation-related variability. Analyses were performed using Cormay reagents (PZ Cormay S.A., Lublin, Poland) and a BS-180 biochemical analyser (Mindray, Shenzhen, China). Total albumin (ALB), lipase (LIPA), urea (UREA) and creatinine (CREA), alkaline phosphatase (ALP), aspartate aminotransferase (AST), and alanine aminotransferase (ALT) were determined. All biochemical procedures were performed in accordance with the methodology described by Szczepanik et al. [24] and in line with the manufacturers’ instructions for the analyser and diagnostic kits. Reference values were based on the ranges provided by the reagent manufacturer.

### 2.6. Analysis of Plasma Cholesterol and Triglycerides

Lithium-heparinised blood was centrifuged (4000× *g*, 10 min) to obtain plasma, which was snap-frozen in liquid nitrogen and stored at −80 °C. This centrifugation force is commonly applied in rabbit blood processing and yields clear plasma suitable for biochemical analyses. Triglycerides, total cholesterol, and HDL cholesterol were determined using Pointe Scientific kits (Pointe Scientific Inc., Canton, MI, USA). LDL cholesterol was calculated using the Friedewald formula: LDL = total cholesterol − HDL − (triglycerides/5). Assay sensitivity and coefficients of variation followed kit specifications.

### 2.7. Determination of Oxidative Stress Biomarkers in Blood Plasma

Peripheral whole blood was collected into lithium-heparinized tubes and centrifuged (4000× *g*, 10 min) to obtain plasma. Antioxidant parameters, including SOD and CAT activities, MDA and GSH concentrations, and total antioxidant capacity (FRAP), were determined following the analytical procedures described by Czech et al. [25]. Special care was taken to minimise oxidative changes during sample handling; plasma samples were rapidly processed and frozen to preserve redox status.

### 2.8. Determining the Concentration of Metals

Samples of muscle, liver, kidney and feed (~5 g) were subjected to wet mineralisation in a perchloric/nitric acid mixture (1:3 *v*/*v*, 24 h), followed by thermal digestion (100–180 °C, 6–7 h). Metals (Zn, Cu, Ni, Mn, Fe, Pb, Cd) were quantified by atomic absorption spectrometry (AAS; Unicam 929), following Agemian et al. [26], with adaptations from Pałka et al. [27]. The detection limit (LOD) of the analytical method was <0.01 mg/kg for the analysed elements. Results were expressed as mg/kg wet weight (tissues) or mg/kg dry weight (feeds). The concentrations of trace elements in the experimental diets and pumpkin seed cake are presented in Table 4.

### 2.9. Statistical Analysis

Statistical analyses were performed using a one-way analysis of variance model according to the following structure: Y_i_ = μ + D_i_ + ε_i_, where Y_i_; is the observed value, μ is the overall mean, D_i_ is the fixed effect of diet, and ε_i_ is the random error term. The individual animal was considered the experimental unit for all analyses. The normality of data distribution was verified using the Shapiro–Wilk test; homogeneity of variances was assessed using Levene’s test. Variables meeting assumptions of normality (biochemical indices, oxidative status, mineral content) were analysed by one-way ANOVA followed by Tukey’s HSD post hoc test, which accounts for multiple comparisons. Non-normally distributed variables (haematological parameters) were assessed using the Kruskal–Wallis test, with pairwise comparisons performed using the Mann–Whitney U test, with appropriate adjustment for multiple comparisons (Bonferroni correction). Results are presented as means ± SEM, to reflect the precision of the estimated means; statistical significance was set at *p* ≤ 0.05. The potential influence of sex and diet × sex interactions was initially tested in an extended model; however, neither factor reached statistical significance (*p* > 0.05). Therefore, these effects were not retained in the final model to avoid unnecessary model overparameterization and to focus on the main effect of diet. No additional random effects were included in the model, as the individual animal was considered the experimental unit. All analyses were conducted using Statistica 13.1 (TIBCO Software Inc., Palo Alto, CA, USA). All statistically significant differences (*p* ≤ 0.05) are reported in the corresponding tables. Sample sizes used in the present study are consistent with those commonly applied in controlled in vivo rabbit nutrition experiments and comply with ethical recommendations for animal research, including the 3Rs principle (reduction), ensuring reliable results while minimizing animal use. No outliers were excluded from the statistical analysis.

## 3. Results

### 3.1. Blood Morphology

Statistical analysis showed that dietary PSC significantly affected several haematological parameters (Table 5). The white blood cell count (WBC) was higher in both PSC-supplemented groups than in the control group (*p* < 0.001). The lymphocyte percentage (LYM) was higher in the control group than in T1 and T2 (*p* < 0.001), whereas the granulocyte percentage (GRA) showed the opposite pattern, with higher values in both PSC groups (*p* < 0.001). The monocyte percentage (MON) was highest in T2 (*p* = 0.007). The red blood cell count (RBC), haemoglobin (HGB), and haematocrit (HCT) increased with dietary PSC inclusion, with the highest values observed in T2 (*p* = 0.002, *p* = 0.022, and *p* = 0.009, respectively). RDW-CV was also higher in T2 than in the other groups (*p* = 0.001). Platelet count (PLT) and plateletcrit (PCT) were highest in T1, followed by T2, and the control group (*p* < 0.001 for both). Platelet distribution width (PDW) was lowest in T1 and highest in T2 (*p* = 0.009). No significant effects of diet were found for MCV, MCH, MCHC, RDW-SD, MPV, or P-LCR (*p* > 0.05).

### 3.2. Blood Biochemistry

Dietary PSC significantly influenced several biochemical parameters (Table 6). Lipase (LIP) activity varied between groups (*p* = 0.019), with the lowest value in T1 and the highest in T2. Renal markers were also affected: urea (UREA) was highest in C and lowest in T1 (*p* = 0.023), whereas creatinine (CREA) was highest in T2 and lowest in C (*p* = 0.035). PSC supplementation significantly reduced triglycerides (TG), total cholesterol (CHOL) and LDL cholesterol (all *p* < 0.001). Although HDL cholesterol did not differ significantly (*p* = 0.549), a slight upward trend with increasing PSC inclusion was observed. No significant effects were detected for albumin (ALB; *p* = 0.466), alanine aminotransferase (ALT; *p* = 0.285) or alkaline phosphatase (ALP; *p* = 0.289), although ALP activity exceeded reference values in all groups. All measured biochemical parameters remained within or close to physiological ranges reported for growing rabbits; however, reference values may vary depending on age and developmental stage.

### 3.3. Oxidative Status

Dietary PSC significantly affected oxidative stress markers (Table 7). Malondialdehyde (MDA) levels decreased with PSC supplementation (*p* < 0.001), while superoxide dismutase (SOD) activity increased across all groups (*p* < 0.001). Ferric-reducing antioxidant power (FRAP) was also enhanced, with the highest values in T2 (*p* = 0.030). In contrast, no significant differences were observed for catalase (CAT) activity (*p* = 0.093) or glutathione (GSH) levels (*p* = 0.217). Although reference ranges for oxidative stress biomarkers are not well standardised in rabbits and depend on analytical methodology and physiological status, the observed changes are consistent with an improvement in antioxidant capacity rather than a disruption of redox homeostasis.

### 3.4. Metal Levels in Tissues

Overall, dietary PSC had a limited effect on metal accumulation in tissues, with significant differences observed mainly for manganese and selected elements in internal organs. The mineral composition of the experimental diets and pumpkin seed cake is presented in Table 4.

Among the analysed metals in muscle tissue, a significant difference between groups was observed only for manganese (Mn) (*p* = 0.001; Table 8), with higher concentrations in the control group and lower values in both PSC-supplemented groups (T1 and T2). No other metals differed significantly between treatments. Lead (Pb) concentrations were below the detection limit of the analytical method in all muscle samples.

In liver tissue, significant differences were observed for nickel (Ni), cadmium (Cd), and copper (Cu) (Table 9). Nickel concentrations were lowest in T1, while the control and T2 groups showed higher and comparable values (*p* = 0.005). Cadmium levels were highest in T1 and lower in both C and T2 (*p* = 0.018). Copper content also differed between groups (*p* = 0.015), with the highest values in T1 and the lowest in T2, while the control group showed intermediate levels. No significant differences were found for Fe, Zn, or Mn. Lead (Pb) concentrations were below the detection limit of the analytical method in all liver samples.

In kidney tissue, significant differences were observed for cadmium (Cd) and manganese (Mn) (Table 10). Cadmium concentrations were highest in T1 and lower in both C and T2 (*p* = 0.011), while manganese levels were highest in the control group and reduced in both PSC-supplemented groups (*p* = 0.005). No other metals showed significant differences between treatments.

## 4. Discussion

The improvement in haematological parameters observed in rabbits receiving pumpkin seed cake (PSC) can be attributed to the biologically active compounds naturally present in pumpkin seeds [6]. Flavonoids, polyphenols, and tocopherols support erythropoiesis by enhancing iron utilisation and stabilising erythrocyte membranes, which is consistent with the higher RBC, HGB, and HCT values as well as improved RBC indices (e.g., MCHC) in PSC-fed rabbits [28]. Furthermore, phytosterols and unsaturated fatty acids may stimulate leukocyte proliferation and modulate inflammatory pathways, aligning with the increased WBC and granulocyte counts observed in the treatment groups [29]. However, an increase in WBC and granulocyte counts may also reflect physiological immune activation rather than exclusively beneficial stimulation [30]. In the present study, as these changes were not accompanied by a deterioration in biochemical or oxidative stress parameters, they are more likely to indicate adaptive immunomodulation rather than a stress-related response. PSC also supplies high-quality protein and micronutrients that support normal haematopoiesis, contributing to the overall improvement in blood morphology [31]. Studies by Ibrahim et al. [32] showed increases in red blood cell (RBC), white blood cell (WBC), and haemoglobin (HGB) levels in rabbits, likely due to the stimulation of haematopoiesis by flavonoids and polyphenols present in the seeds. Similarly, in our study, PSC improved RBC quality (MCHC) and increased WBC counts. Njidda and Isidahomen [33] also reported an increase in WBC and HGB in response to sesame seed meal supplementation, attributed to the regulatory effects of lignans and tocopherols on erythropoiesis. These findings are consistent with our results, suggesting that pumpkin seed cake exerts a positive effect on haematopoiesis and immune function.

A number of studies have been conducted to examine the effects of pumpkin-derived products on rabbit haematology. Ragab et al. [34] reported that supplementation with pumpkin seed oil or black cumin oil did not significantly alter blood parameters, likely because pressed oils contain fewer bioactive compounds than oilcake, which is richer in protein, fibre and phytochemicals. In contrast, the present study demonstrated clear haematological improvements after PSC supplementation, suggesting a higher biological value of the unprocessed cake. In a study conducted by Abdelnour et al. [35], it was observed that rabbits fed a diet containing pumpkin seed oil exhibited increased levels of haemoglobin (HGB) and decreased levels of white blood cells (WBC) in response to heat stress. This suggests a potential protective effect of pumpkin seed oil under such conditions. Differences between these studies and ours likely reflect the distinct composition of oil versus oilcake, with the latter providing additional immunomodulatory components. Ekpo et al. [36] reported that adding 10% pumpkin stem waste increased HGB and RBC but reduced MCH and MCV, suggesting the production of smaller, less haemoglobin-rich erythrocytes. Their supplement did not affect WBC, whereas PSC increased WBC, RBC, and MCHC, indicating improved erythrocyte quality and immune status. These differences between studies likely reflect variation in the form and composition of pumpkin-derived ingredients, particularly in the higher content of protein and bioactive compounds in oilcake compared with other plant fractions. Accordingly, the haematological response to PSC may depend not only on the presence of bioactive compounds, but also on the form of the pumpkin-derived ingredient and its overall nutrient composition. Importantly, all haematological values remained within physiological reference ranges for growing rabbits, suggesting that PSC supplementation induced adaptive rather than pathological changes and may beneficially modulate haematological parameters.

Biochemical blood parameters, assessed in plasma, provide essential information about the animal’s metabolic and health status [37]. In the present study, pancreatic lipase activity, a hallmark of digestive and pancreatic function [38], remained within standard limits despite observed disparities between groups, indicating no adverse impact on fat digestion. Creatinine and urea concentrations, which are frequently utilised as markers of renal function [39,40], also remained within the reference range, indicating that PSC supplementation did not impair kidney function. Slight increases in creatinine may also be associated with higher dietary protein utilisation or muscle turnover rather than renal dysfunction, particularly in growing animals [41]. Although creatinine was higher in the 10% PSC group, all values remained within the physiological reference range for rabbits, suggesting that this increase reflects normal variation in protein and muscle metabolism rather than impaired kidney function. A gradual increase in creatinine values with increasing PSC inclusion was also observed, suggesting a dose-dependent effect. No significant effects were detected for alkaline phosphatase (ALP), although its activity exceeded commonly reported reference ranges in all groups. Despite the lack of statistical significance, elevated ALP activity in growing rabbits is often associated with intensive bone development and increased osteoblastic activity rather than pathological processes, which may explain the values observed in the present study [42]. Moreover, the lack of significant changes in other biochemical indicators suggests that the observed ALP activity is unlikely to be associated with pathological conditions. The analysis of the lipid profile indicated evident dietary influences on triglycerides, total cholesterol, and LDL fractions, which are pivotal indicators of lipid metabolism and cardiovascular risk [43]. The observed improvements confirm that PSC can beneficially modulate lipid homeostasis. The results of the present study suggest that PSC does not adversely affect metabolic status under the conditions of the present study and may contribute to improved metabolic responses. Bergeron et al. [44] reported that replacing soy protein with fish protein increased total cholesterol and LDL levels in rabbits, despite lowering triglycerides, suggesting that dietary protein sources can markedly influence lipid metabolism. In contrast, PSC reduced both total cholesterol and LDL, indicating a more favourable hypolipemic effect than fish protein. Abdl-Rahman et al. [45] showed that adding urea to rabbit diets elevated ALT, urea, and creatinine, reflecting metabolic strain on the liver and kidneys. The reduction in urea in PSC-fed rabbits suggests a protective effect on renal metabolism. Etim and Oguike [46] observed non-significant increases in albumin levels after supplementation with wild sunflower, which aligns with our findings that PSC did not significantly affect albumin concentration.

A number of studies have evaluated the potential of pumpkin-derived feed components in relation to rabbit lipid metabolism. Ragab et al. [34] demonstrated that the ingestion of pumpkin seed oil and black cumin oil resulted in a reduction in total cholesterol and triglyceride levels, although they concomitantly observed a decrease in HDL levels. The observed effects were attributed to the presence of polyunsaturated fatty acids, phytosterols, and dietary fibre, which may influence lipid metabolism pathways and cholesterol absorption. The present study corroborates the hypolipidemic potential of pumpkin-derived ingredients, as PSC diminished total cholesterol, LDL, and triglycerides. However, in contrast to oil-based supplements, HDL exhibited an upward trend. In a similar manner, Ekpo et al. [36] demonstrated that pumpkin greens were capable of reducing total cholesterol, LDL, and triglycerides, whilst concomitantly increasing HDL. This effect was hypothesised to be a consequence of the fibre content and bioactive compounds of the pumpkin greens. Bakeer [47] reported enhanced pancreatic enzyme activity after pumpkin seed oil supplementation, suggesting improved digestive function. Abou-Shehema et al. [48] also observed significant reductions in total cholesterol, LDL, and triglycerides with pumpkin seed meal. These findings are consistent with the results of the present study, although PSC did not have a significant effect on albumin. Taken together, these findings suggest that the hypolipidemic effect observed in the present study is more likely related to the combined action of PSC-derived fibre, unsaturated fatty acids, and antioxidant compounds than to a single bioactive component. Overall, the lipid-lowering effects observed in this study support the potential of pumpkin seed cake as a functional ingredient contributing to improved lipid metabolism and maintenance of metabolic homeostasis in rabbits. These effects should be interpreted in the context of PSC inclusion rather than in the direct replacement of soybean meal, as differences in amino acid composition and energy value between diets may have contributed to the observed responses. While the present study focuses on physiological and metabolic responses, it should be noted that growth performance and feed intake were evaluated within the same experimental trial and are reported separately [22]. Therefore, potential links between physiological changes and production performance require integrated analysis and should be interpreted with caution.

Oxidative stress reflects an imbalance between pro-oxidant processes and antioxidant defences, leading to cellular damage [49,50]. In our study, PSC supplementation significantly reduced MDA levels and increased SOD activity and FRAP values, indicating enhanced antioxidant status. This effect may be related to the presence of natural antioxidants in PSC, such as tocopherols, phenolic compounds and carotenoids, which may contribute to improved redox balance by enhancing enzymatic antioxidant defence (e.g., SOD activity) and supporting non-enzymatic antioxidant capacity (FRAP) [51]. Balkan et al. [52] showed increased MDA and reduced GSH in rabbits fed a cholesterol-rich diet, whereas PSC lowered MDA without affecting GSH, suggesting that PSC may act predominantly at early stages of the antioxidant response. Sikiru et al. [53] demonstrated that chlorella supplementation decreased MDA and increased antioxidant enzymes due to its vitamin and carotenoid content. A similar mechanism may apply to PSC, which contains natural tocopherols and β-carotene. Mattioli et al. [54] reported improved plasma antioxidant capacity and reduced DNA damage after olive leaf and selenium supplementation, reflecting synergistic antioxidant mechanisms. These findings are consistent with previous studies [55] demonstrating that pumpkin-derived products, such as pumpkin seed oil, can enhance antioxidant status in rabbits by increasing total antioxidant capacity and reducing lipid peroxidation markers such as MDA. Likewise, PSC markedly increased FRAP, particularly at 10% inclusion, indicating enhanced non-enzymatic antioxidant protection. Overall, evidence from the literature and our results confirms that pumpkin seed cake effectively strengthens the antioxidant defence system in rabbits by reducing lipid peroxidation and enhancing enzymatic and non-enzymatic antioxidant capacity. Its selective action—the lack of an effect on CAT and GSH—may reflect the predominance of non-enzymatic antioxidants in PSC and the modulation of early stages of oxidative defence. It is also possible that CAT and GSH systems were not significantly activated because the oxidative status of the animals was not sufficiently challenged to induce their upregulation [56]. Importantly, the observed changes in oxidative stress markers reflect an improvement in redox balance rather than a deviation from physiological homeostasis, indicating a beneficial effect of PSC supplementation.

Heavy metals and trace elements have been demonstrated to play diverse roles in animal physiology. For instance, essential elements, such as Fe, Zn, Cu and Mn, act as enzyme cofactors and participate in oxidative balance and oxygen transport [57]. By contrast, toxic metals, such as Pb and Cd, accumulate, even at low concentrations, and pose health risks. Consequently, the monitoring of these levels in rabbit tissues is imperative for evaluating both animal health and consumer safety. It has been demonstrated by preceding studies that tissue concentrations of metals can be significantly impacted by dietary composition and environmental exposure. As stated by Gaafar et al. [58], the administration of lead (Pb)- and cadmium (Cd)-contaminated feed resulted in a significant accumulation of these metals in the kidneys and liver, confirming the kidney as a sensitive biomarker of Cd exposure. In contrast, Zeweil et al. [59] demonstrated that essential elements, such as Cu, Fe, and Zn, exhibit the highest concentrations in the liver, which serves as the primary storage organ. While muscle concentrations are lower, they are subject to modulation by antioxidants or metal-binding substances present in the diet.

Pałka et al. [27] reported that nettle and fenugreek supplementation reduced iron concentrations in rabbit liver and muscle, likely due to phenolic and chelating compounds limiting Fe absorption. Nickel accumulation was observed to increase in the nettle group, while cadmium was detected exclusively in the liver, with the highest levels also being detected in nettle-fed rabbits. In contrast, PSC did not significantly alter Fe, Cu, Zn, Ni or Cd levels in muscle, although Mn concentrations were significantly reduced, suggesting reduced Mn accumulation. Pogány Simonová et al. [60] demonstrated that enterocin 7420 and sage extract influenced mineral levels in rabbit tissues, particularly iron and zinc, through possible effects on gut microbiota and micronutrient bioavailability. In comparison, the results obtained demonstrated that the control group exhibited the highest levels of Fe, with lower, albeit non-significant, levels observed in rabbits that had been fed a PSC. In a similar manner, the Zn concentrations were found to be highest in the control group, in contrast to the patterns observed with sage and enterocin supplementation. However, it should be noted that these differences did not reach a level of significance. Furthermore, the levels of copper and other trace elements remained unaltered, suggesting that PSC does not significantly impact the homeostasis of these minerals. As demonstrated by Papadomichelakis et al. [61], selenium supplementation led to a reduction in nickel and cadmium levels in rabbit liver, as well as a decrease in copper and cadmium in muscle. This outcome indicates the importance of selenium in the formation of metallo–rotein complexes, which enhance the process of detoxification. In our study, Cd levels remained low in all groups, while Ni was higher in T2 liver, suggesting PSC lacks the chelating potency of selenium. Overall, these comparisons indicate that the effect of PSC on trace element distribution was limited and tissue-specific, suggesting modulation of mineral homeostasis rather than a broad detoxifying effect. From a toxicological perspective, this observation should be interpreted with caution, as even moderate increases in heavy metal accumulation in internal organs may indicate altered metal metabolism or deposition patterns [62]. According to Commission Regulation (EU) 2023/915 [63], the maximum permissible level of cadmium in meat is 0.05 mg/kg. All values in this study were below this threshold, indicating no immediate food safety concern for edible muscle tissue under the conditions of the present experiment. Although edible muscle tissue remained within acceptable limits, the accumulation of metals in internal organs (kidneys) may indicate altered metal metabolism and should be taken into account in long-term risk assessments. Furthermore, although no EU maximum level has been established for nickel in rabbit tissues, the elevated Ni concentrations observed in PSC-fed rabbits warrant further investigation, particularly with regard to long-term exposure. Therefore, while PSC did not indicate acute toxicological risk in this short-term study, the observed changes in Cd and Ni distribution suggest that further long-term studies are needed to fully assess its safety.

One limitation of the present study is that the experimental diets were not strictly isoenergetic or isoaminoacidic. Despite the fact that the diets were formulated to meet the nutritional requirements of growing rabbits and were comparable in crude protein content, it is possible that differences in amino acid composition and energy-related components may have influenced the observed responses. Consequently, the contribution of these dietary differences cannot be completely excluded. Furthermore, pumpkin seed cake was used to partially or completely replace soybean meal in the experimental diets. However, it should be noted that the study was not designed to directly compare protein sources or assess their nutritional equivalence. Consequently, the observed effects should be interpreted as responses to the inclusion of dietary PSC rather than as a direct substitution effect. The experiment was conducted over a defined growth period and on a moderate sample size, which may limit the extrapolation of the results to long-term physiological adaptation or broader production conditions. It is important to note that the oxidative stress parameters were determined in a reduced number of animals. This should also be taken into account when interpreting these findings. Another limitation is that LDL cholesterol was estimated using the Friedewald formula, originally developed for humans and not specifically validated for rabbits. Consequently, LDL values should be interpreted as approximate rather than absolute. Furthermore, only a select number of biomarkers of metabolic and oxidative status were evaluated; the lack of gut microbiota analysis represents an additional limitation, particularly given the key role of intestinal microbiota in rabbit physiology. The nutritional composition of PSC is subject to variation depending on cultivar, agronomic conditions, and processing methods, which may influence its fibre, protein, antioxidant and mineral content. It is recommended that future research include longer feeding trials, larger populations, and a broader range of physiological and production parameters. Further research is required in the form of comparative studies evaluating PSC alongside other protein sources. Such studies should also encompass the effects of PSC on production traits, immunity, and gut health. This would serve to further strengthen the understanding of its role in rabbit nutrition.

## 5. Conclusions

This study demonstrated that dietary inclusion of pumpkin seed cake (PSC) at 5% and 10% did not adversely affect the evaluated physiological parameters and may be considered a promising dietary component within the tested inclusion levels under the conditions of the present study. PSC improved haematological and biochemical profiles, including reduced urea, triglyceride, total cholesterol, and LDL cholesterol concentrations, together with a dose-related increase in creatinine that remained within physiological limits. It also enhanced oxidative status, as shown by decreased MDA and increased SOD activity and FRAP values. PSC reduced manganese accumulation in muscle and kidney tissues, while cadmium and lead concentrations remained within regulatory limits, indicating no immediate food safety concern for edible tissues. However, the observed differences in cadmium and nickel distribution in selected organs suggest that further long-term studies are required to fully assess the toxicological implications of PSC inclusion in rabbit diets. Overall, the results indicate that PSC supports selected physiological and antioxidant responses in rabbits; however, these effects should be interpreted in relation to dietary inclusion levels and not as evidence of direct nutritional equivalence with soybean meal.

## Figures and Tables

**Table 1:** Component composition of experimental diets (%).

Component	Group		
	C	T1	T2
Post-extraction soybean meal	13.00	6.50	0.00
Alfalfa meal	20.10	20.10	22.10
Wheat bran	18.10	20.00	17.00
Ground barley	29.80	33.80	42.00
Ground maize	10.00	6.65	2.00
Rapeseed oil	2.10	1.05	0.00
Dicalcium phosphate	1.00	1.00	1.00
NaCl	0.40	0.40	0.40
Mineral and vitamin premix	1.00	1.00	1.00
Arbocel	4.50	4.50	4.50
Pumpkin seed cake	0.00	5.00	10.00

Notes: C—control; T1—5% PSC; T2—10% PSC.

**Table 2:** Chemical composition of experimental diets and pumpkin seed cake (% of dry matter).

Chemical Composition	Dose			PSC
	C	T1	T2	
Dry matter (%)	90.20	90.04	90.28	92.48
Crude ash (%)	6.02	5.76	5.71	7.12
Crude fat (%)	5.19	5.05	5.45	26.52
Crude protein (%)	16.52	16.31	16.45	43.36
Crude fibre (%)	10.68	11.92	11.16	2.44
NFE (%)	51.79	51.00	51.51	20.56
Estimated DE (MJ/kg DM)	13.08	12.74	13.01	21.98

Notes: C—control; T1—5% PSC; T2—10% PSC; PSC—pumpkin seed cake; NFE—nitrogen-free extract; DE—digestible energy.

**Table 3:** Amino acid composition of experimental diets (g/kg feed).

Amino Acid	Dose		
	C	T1	T2
Aspartic acid (Asp)	15.86	11.55	10.26
Threonine (Thr)	6.32	4.73	4.30
Serine (Ser)	8.43	6.23	5.82
Glutamic acid (Glu)	30.14	24.78	22.82
Proline (Pro)	11.21	9.72	9.27
Glycine (Gly)	7.96	6.34	6.00
Alanine (Ala)	8.28	6.16	5.80
Valine (Val)	8.11	6.33	5.92
Isoleucine (Ile)	6.63	5.07	4.36
Leucine (Leu)	12.58	9.54	8.60
Tyrosine (Tyr)	5.86	4.16	3.31
Phenylalanine (Phe)	8.03	5.86	5.35
Histidine (His)	5.53	2.99	2.63
Lysine (Lys)	9.69	6.43	5.79
Arginine (Arg)	10.00	8.82	8.87
Cysteine (Cys)	2.62	2.44	2.31
Methionine (Met)	2.41	2.20	2.32

**Table 4:** Metal concentrations in pumpkin seed cake and experimental diets (mg/kg dry weight).

Metal	Dose			PSC
	C	T1	T2	
Cu	10.12	11.47	11.98	15.24
Fe	267.86	165.08	144.28	29.09
Zn	88.21	88.05	85.81	78.37
Mn	34.98	22.31	17.87	8.454
Ni	4.285	3.512	3.107	2.949
Pb	0.439	0.190	0.032	0.046
Cd	0.155	0.153	0.136	0.031

Notes: C—control; T1—5% PSC; T2—10% PSC; PSC—pumpkin seed cake.

**Table 5:** Effect of adding pumpkin seed cake on rabbit complete blood count results (mean ± SEM).

Parameter	Group			*p*-Value	Reference Values
	C *n* = 10	T1 *n* = 10	T2 *n* = 10		
WBC (×10^3^ µL)	8.46± 0.70 ^b^	11.74 ± 0.53 ^a^	10.31 ± 0.44 ^a^	<0.001	3–12
LYM (%)	78.54 ± 0.79 ^a^	68.41 ± 2.13 ^b^	69.74 ± 1.62 ^b^	<0.001	25–95
MON (%)	0.84 ± 0.03 ^b^	0.86 ± 0.04 ^b^	1.01 ± 0.05 ^a^	0.007	1–16
GRA (%)	20.63 ± 0.77 ^b^	30.74 ± 2.10 ^a^	29.26 ± 1.59 ^a^	<0.001	10–80
RBC (×10^6^ µL)	5.32 ± 0.05 ^b^	5.45 ± 0.09 ^a^	5.68 ± 0.07 ^a^	0.002	4–9
HGB (g/dL)	11.24 ± 0.10 ^b^	11.48 ± 0.18 ^a^	11.83 ± 0.15 ^a^	0.022	9–19
HCT (%)	33.03 ± 0.30 ^b^	33.86 ± 0.55 ^ab^	34.93 ± 0.40 ^a^	0.009	30–53
MCV (µm^3^)	62.55 ± 0.43	62.12 ± 0.19	61.51 ± 0.218	0.054	60–80
MCH (pg)	21.27 ± 0.15	21.07 ± 0.09	21.37 ± 0.42	0.715	16–30
MCHC (g/dL)	34.02 ± 0.14	33.90 ± 0.10	33.84 ± 0.10	0.533	22–41
RDW-CV (%)	14.41 ± 0.29 ^b^	14.87 ± 0.20 ^b^	15.59 ± 0.15 ^a^	0.001	
RDW-SD (µm^3^)	35.17 ± 0.56	36.68 ± 0.54	37.11 ± 0.48	0.150	
PLT (×10^3^ µL)	182.90 ± 10.19 ^c^	308.35 ± 14.33 ^a^	239.95 ± 12.30 ^b^	<0.001	120–800
MPV (µm^3^)	5.56 ± 0.06	5.68 ± 0.07	5.56 ± 0.10	0.486	
PCT (%)	0.10 ± 0.01 ^c^	0.17 ± 0.01 ^a^	0.13 ± 0.01 ^b^	<0.001	
PDW (%)	21.83 ± 1.39 ^ab^	18.28 ± 0.64 ^b^	23.10 ± 1.20 ^a^	0.009	
P-LCR	4.84 ± 0.28	4.74 ± 0.34	4.63 ± 0.52	0.931	12–42

Notes: C—control; T1—5% PSC; T2—10% PSC. Different superscripts indicate significant differences (*p* ≤ 0.05). SEM—standard error of the mean; *p*-values were calculated using the Kruskal–Wallis test. Abbreviations: WBC—white blood cells; LYM—lymphocytes; MON—monocytes; GRA—granulocytes; RBC—red blood cells; HGB—haemoglobin; HCT—haematocrit; MCV—mean corpuscular volume; MCH—mean corpuscular haemoglobin; MCHC—mean corpuscular haemoglobin concentration; RDW-CV—red cell distribution width (coefficient of variation); RDW-SD—red cell distribution width (standard deviation); PLT—platelets; MPV—mean platelet volume; PCT—plateletcrit; PDW—platelet distribution width; P-LCR—platelet large cell ratio. Reference values were provided by the haematology analyser manufacturer; ranges were not available for all parameters.

**Table 6:** Effect of adding pumpkin seed cake on biochemical parameters of rabbit blood (mean ± SEM).

Parameter	Group			*p*-Value
	C *n* = 10	T1 *n* = 10	T2 *n* = 10	
ALT (U/L)	35.84 ± 5.30	48.11 ± 5.55	44.55 ± 5.63	0.285
ALP (U/L)	173.05 ± 20.87	216.06 ± 23.98	216.30 ± 21.76	0.289
ALB (g/dL)	3.72 ± 0.16	3.48 ± 0.16	3.78 ± 0.20	0.466
LIP (U/L)	251.47 ± 15.28 ^ab^	233.42 ± 23.74 ^b^	330.66 ± 30.46 ^a^	0.019
CREA (mg/dL)	0.85 ± 0.06 ^b^	0.95 ± 0.06 ^ab^	1.06 ± 0.05 ^a^	0.035
UREA (mg/dL)	29.31 ± 1.62 ^a^	22.21 ± 1.59 ^b^	24.67 ± 1.90 ^ab^	0.023
CHOL (mg/dL)	208.30 ± 5.52 ^a^	194.30 ± 2.26 ^b^	181.40 ± 2.16 ^c^	<0.001
TG (mg/dL)	189.30 ± 3.69 ^a^	177.40 ± 3.24 ^b^	161.10 ± 2.72 ^c^	<0.001
HDL (mg/dL)	59.20 ± 2.29	61.00 ± 1.39	62.00 ± 1.63	0.549
LDL (mg/dL)	111.24 ± 3.73 ^a^	97.82 ± 2.09 ^b^	87.18 ± 2.32 ^c^	<0.001

Notes: C—control; T1—5% PSC; T2—10% PSC. Different superscripts indicate significant differences (*p* ≤ 0.05). SEM—standard error of the mean. Abbreviations: ALT—alanine aminotransferase; ALP—alkaline phosphatase; ALB—albumin; LIP—lipase; CREA—creatinine; UREA—urea; CHOL—cholesterol; TG—triglycerides; HDL—high-density lipoprotein; LDL—low-density lipoprotein.

**Table 7:** Effects of pumpkin seed cake on indicators of redox status in rabbit serum (mean ± SEM).

Parameter	Group			*p*-Value
	C *n* = 6	T1 *n* = 6	T2 *n* = 6	
MDA (µmol/L)	2.183 ± 0.07 ^a^	1.577 ± 0.14 ^b^	1.482 ± 0.09 ^b^	<0.001
SOD (U/mL)	23.989 ± 0.09 ^a^	26.586 ± 0.40 ^b^	27.764 ± 0.17 ^c^	<0.001
CAT (U/mL)	19.563 ± 0.63	19.398 ± 0.65	17.575 ± 0.70	0.093
GSH (µmol/L)	4.934 ± 0.03	4.734 ± 0.12	4.915 ± 0.08	0.217
FRAP (µmol/L)	6.246 ± 0.08 ^a^	7.603 ± 0.40 ^ab^	7.736 ± 0.54 ^b^	0.030

Notes: C—control; T1—5% PSC; T2—10% PSC. Different superscripts indicate significant differences (*p* ≤ 0.05). SEM—standard error of the mean. Abbreviations: MDA—malondialdehyde; SOD—superoxide dismutase; CAT—catalase; GSH—glutathione; FRAP—ferric-reducing antioxidant power.

**Table 8:** Effect of pumpkin seed cake on the metal content of rabbits’ longissimus lumborum muscle (mg/kg wet weight) (mean ± SEM).

Metal (mg/kg)	Group			*p*-Value
	C *n* = 10	T1 *n* = 10	T2 *n* = 10	
Cu	0.62 ± 0.04	0.56 ± 0.04	0.60 ± 0.03	0.548
Fe	4.69 ± 0.57	3.46 ± 0.19	3.88 ± 0.19	0.070
Zn	9.38 ± 0.46	8.25 ± 0.61	8.08 ± 0.31	0.128
Mn	0.07 ± 0.01 ^a^	0.03 ± 0.01 ^b^	0.03 ± 0.01 ^b^	0.001
Ni	0.93 ± 0.05	1.10 ± 0.08	1.06 ± 0.12	0.373
Pb	<LOD	<LOD	<LOD	-
Cd	0.01 ± 0.01	0.01 ± 0.01	0.01 ± 0.01	0.445

Notes: C—control; T1—5% PSC; T2—10% PSC. Different superscripts indicate significant differences (*p* ≤ 0.05). SEM—standard error of the mean. LOD—limit of detection. Pb values were below the detection limit of the method (<0.01 mg/kg).

**Table 9:** Effect of pumpkin seed cake on the metal content of rabbit liver (mg/kg wet weight) (mean ± SEM).

Metal (mg/kg)	Group			*p*-Value
	C *n* = 10	T1 *n* = 10	T2 *n* = 10	
Cu	4.52 ± 0.40 ^ab^	5.12 ± 0.46 ^a^	3.49 ± 0.20 ^b^	0.015
Fe	35.84 ± 4.74	48.08 ± 7.35	45.51 ± 7.36	0.391
Zn	28.21 ± 0.92	29.65 ± 2.22	30.61 ± 1.09	0.506
Mn	0.71 ± 0.05	0.73 ± 0.05	0.72 ± 0.08	0.968
Ni	1.16 ± 0.062 ^ab^	0.96 ± 0.07 ^b^	1.30 ± 0.06 ^a^	0.005
Pb	<LOD	<LOD	<LOD	-
Cd	0.03 ± 0.01 ^a^	0.05 ± 0.001 ^b^	0.04 ± 0.01 ^ab^	0.018

Notes: C—control; T1—5% PSC; T2—10% PSC. Different superscripts indicate significant differences (*p* ≤ 0.05). SEM—standard error of the mean. LOD—limit of detection. Pb values were below the detection limit of the method (<0.01 mg/kg).

**Table 10:** Effect of pumpkin seed cake on the metal content of rabbit kidneys (mg/kg wet weight) (mean ± SEM).

Metal (mg/kg)	Group			*p*-Value
	C *n* = 10	T1 *n* = 10	T2 *n* = 10	
Cu	2.44 ± 0.08	2.46 ± 0.10	2.19 ± 0.09	0.088
Fe	27.61 ± 2.90	21.32 ± 1.01	22.01 ± 1.28	0.055
Zn	17.61 ± 1.19	18.62 ± 0.46	18.14 ± 0.48	0.663
Mn	1.00 ± 0.06 ^a^	0.78 ± 0.04 ^b^	0.75 ± 0.05 ^b^	0.005
Ni	0.96 ± 0.07	1.49 ± 0.41	1.45 ± 0.25	0.082
Pb	0.12 ± 0.07	0.16 ± 0.08	0.13 ± 0.09	0.952
Cd	0.12 ± 0.01 ^b^	0.20 ± 0.03 ^a^	0.12 ± 0.01 ^b^	0.011

Notes: C—control; T1—5% PSC; T2—10% PSC. Different superscripts indicate significant differences (*p* ≤ 0.05). SEM—standard error of the mean.

## Data Availability

The data presented in this study are available from the corresponding author on reasonable request.

## Abbreviations

The following abbreviations are used in this manuscript:

ALB	Albumin
ALP	Alkaline phosphatase
ALT	Alanine aminotransferase
CAT	Catalase
Cd	Cadmium
CHOL	Total cholesterol
CREA	Creatinine
Cu	Copper
Fe	Iron
FRAP	Ferric-reducing antioxidant power
GRA	Granulocytes
GSH	Glutathione
HCT	Haematocrit
HDL	High-density lipoprotein
HGB	Haemoglobin
LDL	Low-density lipoprotein
LIP	Lipase
LYM	Lymphocytes
MCH	Mean corpuscular haemoglobin
MCHC	Mean corpuscular haemoglobin concentration
MCV	Mean corpuscular volume
MDA	Malondialdehyde
Mn	Manganese
MON	Monocytes
MPV	Mean platelet volume
Ni	Nickel
Pb	Lead
PCT	Plateletcrit
PDW	Platelet distribution width
PLT	Platelets
PSC	Pumpkin seed cake
P-LCR	Platelet large cell ratio
RBC	Red blood cells
RDW-CV	Red cell distribution width (coefficient of variation)
RDW-SD	Red cell distribution width (standard deviation)
SEM	Standard error of the mean
SOD	Superoxide dismutase
TG	Triglycerides
UREA	Urea
WBC	White blood cells
Zn	Zinc
